# Raw agro-industrial orange peel waste as a low cost effective inducer for alkaline polygalacturonase production from *Bacillus licheniformis* SHG10

**DOI:** 10.1186/2193-1801-3-327

**Published:** 2014-06-30

**Authors:** Amira M Embaby, Aliaa A Masoud, Heba S Marey, Nadia Z Shaban, Tayssir M Ghonaim

**Affiliations:** Department of Biotechnology, Institute of Graduate Studies and Research, University of Alexandria, 163 Horreya Avenue, P.O. Box 832, Chatby, 21526 Egypt; Faculty of Science, Department of Biochemistry, University of Alexandria, Alexandria, Egypt; Department of Environmental Studies, Institute of Graduate Studies and Research, University of Alexandria, Alexandria, Egypt

**Keywords:** Raw orange peel waste, *Bacillus licheniformis* SHG10, Alkaline polygalacturonase, Low cost effective co-inducer/sole carbon source and statistical sequential optimization

## Abstract

The current study underlines biotechnological valorization of the accumulated and the non-efficiently utilized agro-industrial orange peel waste to produce polygalacturonase (PGase), an industrially important enzyme with augmented demands in enzymes markets, from *Bacillus licheniformis* SHG10. Sequential statistical optimization of PGase production was performed through one variable at a time (OVAT) approach, Plackett-Burman (PB) and response surface methodology (RSM). The impact of introduction of six raw agro-industrial wastes (orange, lemon, banana, pomegranate, artichoke peel wastes and wheat bran) and other synthetic carbon sources separately into the fermentation broth on PGase productivity was studied through OVAT approach. Orange peel waste as sole raw carbon source in basal medium proved to be the best PGase inducer. It promoted PGase productivity with relative specific activity of 166% comparable with the effect imposed by synthetic citrus pectin as a reference inducer. Three key determinants (orange peel waste, pH of the production medium and incubation temperature) had RSM optimal levels of 1.76% (w/v), 8.0 and 37.8°C, respectively along with maximal PGase level (2.69 μg galacturonic acid. min^-1^. mg^-1^) within 48 hrs. Moreover, SHG10 PGase exhibited activity over a wide range of pH (3-11) and an optimal activity at 50°C. Data greatly encourage pilot scale PGase production from *B. licheniformis* SHG10.

## Background

Citrus fruit is one of the commercial crops in the Egyptian market (Mohamed et al. 2010). Orange juice is one of the most consumed beverages today (Martin et al. 2010). Consequently, a high percentage of citrus fruit is used for manufacturing of juice and marmalade. Approximately 50-60% of citrus fruit is transformed into citrus peel waste (Wilkins et al. 2007). This results in accumulation of large quantities of citrus peel waste as a by-product in citrus-processing industry. Accumulated large quantities of the orange peel waste along with environmental considerations to avoid health hazards derived from unsatisfactory disposal methods addressed the indispensable need for finding alternative biotechnological solutions for waste valorization (Martín et al. 2013; Rivas et al. 2008). According to current environmental legislation, any waste could be considered as raw material as long as there is an option to develop method for its valorization (Möller et al. 2001). High value products could be manufactured upon using orange peel waste as a potentially valuable low cost resource (Martin et al. 2010; Rivas et al. 2008; Balu et al. 2012). Orange peel waste was reported to contain 16.9% soluble sugars, 9.21% cellulose, 10.5% hemi-cellulose, and 42.5% pectin as the most important components (Rivas et al. 2008). A vast number of promising methods encountered in efficient utilization of orange peel waste has been described thoroughly in the literature. Among these methods is employing this waste in enzymes industries (Siles and Thompson 2010).

The term pectinolytic enzymes (pectinases) is the generic name of a family of enzymes involved in the process of pectin degradation. This complex bioprocess is achieved mainly by a set of pectinolytic enzymes catalyzed reactions (e.g., hydrolysis, trans-elimination and de-esterification) of the ester bond between the carboxyl and the methyl ester groups of pectin (Rehman et al. 2012). Four types (polygalacturonases, pectin lyases, pectate lyases and pectin methyl esterases) of these enzymes classified based on their mode of action are involved under the generic name of this family (Alkorta et al. 1998; Hoondal et al. 2002; Kuhad et al. 2004). These enzymes have a vast number of industrial applications in food (e.g., juice clarification, refinement of vegetables fibers, extraction of vegetables oil, curing of coffee and cocoa beans) (Silva et al. 2002; Gummadi and Panda 2003; Demir et al. 2012; Quattara et al. 2008; Pedrolli et al. 2009). biopulping of papers (Sittidilokratna et al. 2007) and textiles (Basu et al. 2009). Among pectinolytic enzymes, polygalacturonases (PGase), are the enzymes of particular interest to industry. Endo- PGase (E.C. 3.2.1.67) and exo- PGase ((E.C. 3.2.1.82) catalyze the hydrolysis of internal and external α-1,4 glycosidic bond linking α–galacturonic acid residues in pectin, respectively producing shorter pectin molecular structures, decreasing the viscosity, increasing the yield of juices, and determining the crystalline structure of the final product (Souza et al. 2003).

The up to date review of literature reported a vast number of microorganisms as PGase producers mainly fungi such as *Aspergillus spp*, *Rhizopus stolonifer*, *Alternaria mali*, *Fusarium oxysporum*, *Neurospora crassa*, *Penicillium italicum* ACIM F-152 and a little bit form bacteria confined to *Agrobacterium tumefaciens*, *Bacteroides thetaiotamicron*, *Ralstonia solanacearum, Bacillus spp* and *Enterobacter aerogenes* NBO2 (Jayani et al. 2005, 2010; Darah et al. 2013). Microbial pectinase(s) particularly those of fungal origin account for 25% of the global food and industrial enzymes sales (Demir et al. 2012). Although fungi are considered to be potent pectinases producers, but the drawbacks included in the physicochemical properties of these enzymes greatly limit the utilization of these enzymes on a wide industrial scale (Soares et al. 1999).

The underlying reasons behind carrying out the current study could be outlined in the following annotations; a) increased demand for commercial PGase(s) in the enzymes markets worldwide, b) indispensable need for valorization of the accumulated and the non-efficiently utilized raw agro-industrial orange peel waste in a biotechnological manner and c) necessity for continuous searching for novel PGase(s) with new characteristics to overcome the shortcomings involved in PGase(s) of fungal origin that greatly limit their utilization on a wide industrial scale. In this context, the present study aims to address sequential statistical optimization of PGase production from *Bacillus licheniformis* SHG10 strain upon using the raw agro-industrial orange peel waste as a sole PGase inducer and a sole carbon source in a very low cost effective medium.

## Results

### OVAT results

The influence of different agro-industrial wastes (e.g., orange, lemon, pomegranate, banana, artichokes peel wastes and wheat bran) and synthetic carbon sources (e.g., citrus pectin, glucose, fructose, maltose, xylose, glycerol, sucrose, peptone, beef extract and tryptone) on PGase production by *B. licheniformis* SHG10 strain was studied. Table 1 revealed that orange peel waste at a concentration of 1% (w/v) was the best co-inducer sole carbon source. It enhanced the production of PGase with a relative specific activity of 166% when compared to the effect of citrus pectin (0.5% w/v). While, the substitution of orange peel waste with lemon peel waste (1% w/v) resulted in improvement in PGase level with relative specific activity of 133% of that induced by citrus pectin. However, addition of wheat bran [(0.5% (w/v) and 1% (w/v)] or banana peel waste (1% w/v) separately instead of citrus pectin achieved a level of PGase almost quite similar to that obtained upon using citrus pectin. Conversely, other carbon sources alternative to citrus pectin such as pomegranate and artichoke peel wastes, glucose, fructose, maltose, xylose, sucrose, glycerol, peptone, beef extract and tryptone at the concentrations mentioned in Table 1 exerted suppressive effects on the productivity of PGase by *B. licheniformis* SHG10.

**Table 1:** **Agro-industrial wastes and some synthetic carbon sources as PGase inducers**

Parameter	Relative specific activity (%) ^a,b^
**Agro-industrial wastes (1% w/v):**	
Orange peel waste	**166.80**
Lemon peel waste	133.56
Banana peel waste	105.80
Artichoke peel waste	55.17
Pomegranate peel waste	53.59
**Synthetic carbon sources:**	
Citrus pectin (0.5% w/v)	100.00
Wheat bran (0.5% w/v)	102.00
Wheat bran (1% w/v)	92.00
Glucose (0.5% w/v)	41.82
Fructose (0.5% w/v)	64.10
Maltose (0.5% w/v)	33.01
Xylose (0.5% w/v)	59.04
Sucrose (0.5% w/v)	25.60
Glycerol (0.5% w/v)	27.59
Peptone (0.5% w/v)	33.42
Peptone (1% w/v)	50.20
Beef extract (0.5% w/v)	14.11
Beef extract (1% w/v)	14.11
Tryptone (0.5% w/v)	39.45
Tryptone (1% w/v)	28.72

^a^Specific activity (U/mg) was determined as μg galacturonic acid .min^-1^. mg protein^-1^.

^b^All relative specific activities of PGase were related to U/mg PGase obtained upon using citrus pectin – based basal medium as production medium.

Moreover, the effect of addition of some salts as supplementary substances to the production medium was tested. Data of Table 2 demonstrated that separate introduction of NaNO_3_, KNO_3_, CaCl_2_, FeSO_4_ and MgSO_4_ salts to the control production medium (citrus pectin based- basal medium) each at a concentration of 0.2% (w/v) exhibited diverse degrees of enhancement in the level of PGase with relative specific activity of 145%, 163.8%, 198,9%, 228.57% and 237.7%, respectively when compared to that level obtained upon using control production medium. The addition of (NH_4_)_2_SO_4_ at a final concentration of 0.2% (w/v) resulted in a level of PGase almost equivalent to that of the control production medium. On the other hand, a low level of PGase was detected in the production medium containing NH_4_Cl at a concentration of 0.2% (w/v).

**Table 2:** **Effect of some medium supplements in citrus pectin based- mineral medium on PGase productivity**

Parameter	Relative specific activity (%) ^a,b^
Control medium^c^	100.00
**Salts (0.2% w/v):**	
NaN0_3_	145.00
KN0_3_	163.80
MgS04	237.70
CaCl_2_	198.80
FeS0_4_	228.57
(NH_4_)_2_S0_4_	103.60
NH_4_Cl	72.41
**Yeast extract (0.5%)**	60.21

^a^Specific activity (U/mg): calculated as μg galacturonic acid .min^-1^. mg protein^-1^.

^b^All relative specific activities of PGase were related to U/mg PGase obtained upon using citrus pectin –based basal medium as production medium.

^c^Citrus pectin based- basal medium composition as mentioned in materials and methods.

Concerning the effect of agitation speed on PGase production, three different agitation speeds (100, 150 and 200 rpm) were studied. The level of PGase obtained upon conducting the fermentation process at 150 rpm was higher than that obtained upon conducting the process at 100 rpm (Data not shown). Whereas, the level of PGase obtained upon conducing the fermentation process at 150 rpm was comparable with that obtained upon conducting the process at 200 rpm (Data not shown). Accordingly, agitation speed at 150 rpm was selected to carry out further optimization experiments.

### PBD results

**Figure 1:**
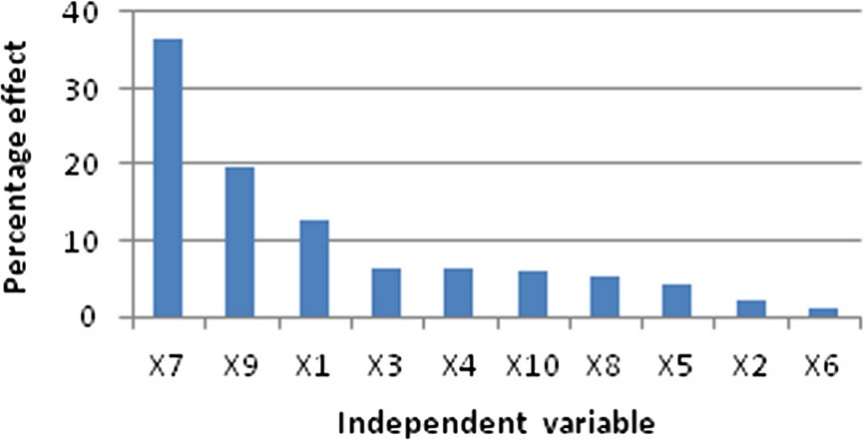
**Pareto-Chart in descending layout for PB parameter estimates of ten tested independent variables.**

PBD matrix, coded-real values of independent variables and experimental vs. predicted values of PGase were shown in Table 3. Whilst regression analysis and independent variables evidenced significant consequences on PGase levels were presented in Table 4. The detected PGase activity ranged from 0.0 to 3.04 U/ml reflecting the irreplaceable necessitate for carrying out optimization in order to attain the possible highest levels of PGase. ANOVA results showed that the P-value and F-value of the model were 0.00087 and 10.26, respectively. This F-value 10.26112 of the model reflects the significance of the model. However, the model P-value implies that the chance is only 0.087% that this model F-value could occur due to noise. Values of “Prob. > *F*” less than 0.05 were taken into account to have significant substantial effect on the outcome. Generally, significance of the coefficients has been reported to be directly proportional to t-test and inversely to P-value (Douglas 2001; Heck et al. 2005). Regression analysis suggested that the level of PGase was significantly affected by only three independent variables out of ten tested independent variables. These three independent variables showing significant effects at P < 0.05 were orange peel waste percent, pH of the production medium and incubation temperature. Pareto chart (Figure 1) is a convenient way to illustrate the order of significance of independent variables affecting PGase production based on their P-values. After exclusion of the insignificant model terms (based on their insignificant P-values >0.05), a modified first order polynomial equation (1) was set in terms of coded independent variables in order to describe the linear effects of orange peel waste percent, pH of the production medium and incubation temperature on PGase level.

**Table 3:** **PBD with coded levels of ten independent variables and experimental vs. predicted PGase values**

Trial#	X _1_	X _2_	X _3_	X _4_	X5	X _6_	X _7_	X _8_	X _9_	X _10_	Y (PGase) (U/mL) ^c^	
											Exp ^a^.	Pred ^b^.
1	1	-1	1	1	1	1	-1	-1	1	1	**0.00**	0.1585
2	-1	1	-1	1	1	1	1	-1	-1	1	1.60	1.6135
3	-1	-1	1	-1	1	-1	1	1	1	1	0.64	1.0695
4	1	1	-1	-1	1	1	-1	1	1	-1	0.15	0.0195
5	1	1	1	1	-1	-1	1	1	-1	1	2.70	2.3865
6	-1	-1	-1	1	-1	1	-1	1	1	1	0.00	-0.5795
7	1	-1	1	-1	1	1	1	1	-1	-1	**3.04**	2.6755
8	-1	1	-1	1	-1	1	1	1	1	-1	0.62	1.0835
9	-1	1	1	-1	-1	-1	-1	1	-1	1	0.00	0.1175
10	-1	-1	-1	-1	1	-1	1	-1	1	1	0.78	0.5935
11	1	1	-1	-1	-1	-1	1	-1	1	-1	1.04	1.0895
12	-1	-1	-1	-1	-1	-1	-1	-1	-1	-1	0.24	-0.0305
13	1	-1	-1	-1	-1	1	-1	1	-1	1	0.00	0.4895
14	-1	1	1	1	1	-1	-1	1	1	-1	0.15	-0.0145
15	1	1	1	-1	-1	1	1	-1	1	1	1.37	1.1425
16	-1	-1	1	1	-1	1	1	-1	-1	-1	1.94	2.0235
17	1	-1	-1	1	1	-1	1	1	-1	-1	2.58	2.6325
18	1	-1	1	1	-1	-1	-1	-1	1	-1	0.00	0.1875
19	-1	1	1	-1	1	1	-1	-1	-1	-1	0.27	0.3635
20	1	1	-1	1	1	-1	-1	-1	-1	1	0.49	0.5885

^a^Experimental values, ^b^predicted values, ^**c**^U/mL: calculated as μg galacturonic acid.min^-1^.mL^-1^.

X_1_: orange peel waste, X_2_: NaNO_3_, X_3_: MgSO_4_, X_4_: CaCl_2_, X_5_: FeSO_4_, X_6_: KNO_3_, X_7_: pH, X_8_: inoculum size, X_9_: incubation temperature and X_10_: incubation time.

**Table 4:** **Real-coded values of ten independent variables and regression analysis for PGase optimization by PBD**

Variable	Low level	High level	Main effect	*Β*-coefficient	P-value	t-value	% confidence
	-1	+1					
**Orange peel waste (% w/v)**	0.5	2.0	0.513	0.2565	0.020839*	2.796274	**97.92**
NaNO_3_ (% w/v)	0.1	0.4	-0.083	-0.0415	0.661675	-0.45242	33.84
MgSO_4_ (% w/v)	0.1	0.4	0.261	0.1305	0.188555	1.422666	81.15
CaCl_2_ (% w/v)	0.1	0.4	0.255	0.1275	0.197956	1.389961	80.21
FeSO_4_ (% w/v)	0.1	0.4	0.179	0.0895	0.354709	0.975698	63.53
KNO3 (% w/v)	0.1	0.4	0.037	0.0185	0.844651	0.201681	15.45
**pH**	6.0	9.0	1.501	0.7505	1.85E-05*	8.181689	**99.99**
Inoculum size (% v/v)	1.0	5.0	0.215	0.1075	0.271314	1.171928	72.87
**Incubation temperature°C**	37.0	50.0	-0.811	-0.4055	0.00167*	-4.42062	**99.83**
Incubation time (hrs)	24.0	60.0	-0.245	-0.1225	0.214517	-1.33545	78.55

*Significant P-value <0.05 & R^2^ = 0.93 & Adjusted R^2^ = 0.83 and P -value for the model = 0.000869.

1

These three chosen independent variables recognized by PB were considered to be the main significant key determinants for PGase production by *B. licheniformis* SHG10. They were further studied in the next stage of the optimization plan via RSM.

### RSM results

Box-Behnken design, a kind of RSM approach, was employed in this study in order to locate the optimal level of the three independent key determinants identified through PB that controlling PGase production. The design matrix along with the experimental and predicted PGase levels were displayed in Table 5. ANOVA results showed that the model F-value of 24.3 and the model P-value of 0.0013 imply the significance of the model and the likelihood (0.13%) that this F-value could occur due to noise. Moreover, adequacy of the model to explain the relationship between the response (output) and the significant independent variables could be measured by the small model P-value 0.0013 and the large lack of fit P-value of 0.276. The lack of fit F-value (2.77) indicates that it is not significant relative to the pure error. Non-significant lack of fit reflects goodness of the model. Aptness of the model was inferred from the R^2^ value of 0.977. Regression coefficients were calculated in terms of coded values of independent variables and data were fitted to a second order polynomial equation (2).

**Table 5:** **Box-Behnken design of three independent variables and experimental vs. predicted PGase values**

Trial#	X _1_	X _7_	X _9_	Y (PGase) (U/mg) ^c^	
				Exp ^a^.	Pred ^b^.
1	-1	-1	0	0.85	0.767
2	1	-1	0	2.03	2.052
3	-1	1	0	2.27	2.247
4	1	1	0	1.15	1.232
5	-1	0	-1	1.79	1.708
6	1	0	-1	2.22	2.033
7	-1	0	1	0.88	1.066
8	1	0	1	0.93	1.011
9	0	-1	-1	0.823	0.987
10	0	1	-1	1.17	1.273
11	0	-1	1	0.215	0.111
12	0	1	1	0.65	0.485
13	0	0	0	2.48	2.640
14	0	0	0	2.68	2.640
15	0	0	0	2.76	2.640

^a^Experimental values.

^b^Predicted values.

^**c**^U/mg: calculated as μg galacturonic acid.min^-1^.mg^-1^.

X_1_: orange peel waste percent, X_7_: pH of the production medium and X_9_: incubation temperature.

2

Our data revealed that only four out of nine model terms exhibited significant effect (P < 0.05) on PGase production (Table 6). The independent variable incubation temperature showed both linear and quadratic effect at P-values of 0.0023 and 0.00022, respectively. Dissimilarly, the independent variable pH of production medium exhibited both linear and cross interacted effect with the independent variable orange peel waste at P- values of 0.00039 and 0.0026, respectively.

**Table 6:** **Real–coded values of independent variables and regression analysis for PGase optimization by Box-Behnken**

Variable	Low level	Middle level	High level	Model term	Main effect	*Β-*coefficient	t-value	P-value	% confidence
	-1	0	+1						
Orange peel waste (w/v %)	0.5	1.5	2.5	X_1_	0.135	0.0675	0.92151	0.399067	60.10
pH	6.0	8.0	10.0	X_7_	0.3306	0.16525	2.255994	0.073721	92.63
Incubation temp. °C	30.0	40.0	50.0	X_9_	-0.832	-0.416	-5.67923	0.002357*	**99.76**
				X_1_^2^	-0.325	-0.16225	-1.50482	0.192706	80.73
				X_7_^2^	-1.805	-0.90275	-8.37274	0.000398*	**99.96**
				X_9_^3^	-2.044	-1.02275	-9.48571	0.00022*	**99.99**
				X_1_.X_7_	-1.15	-0.575	-5.55072	0.002608*	**99.73**
				X_1_.X_9_	-0.19	-0.095	-0.91708	0.401174	60.00
				X_7_.X_9_	0.044	0.022	0.212375	0.840201	16.00

*Significant P-value <0.05 & R^2^ = 0.977 & Adjusted R^2^ = 0.93 and P -value for the model = 0.0013.

In order to attain the optimized conditions, canonical analysis was carried out. Studying the overall shape of the response and determining whether the stationary point is maximum, minimum or saddle point could be achieved through canonical analysis. Shape of the response is characterized by eigen-values and eigenvectors in the matrix of second order. Directions of principle orientation for the surface are determined by eigenvectors, while signs and magnitude of eigen-values point for surface shape in these directions. Two rules of thumb explaining the concept of eigen-values and their mathematical indications were reported previously (Myers 1976). The 1^st^ rule states that upward and downward curvatures of the response are evidenced by positive and negative eigen-values, respectively. While the 2^nd^ rule states that the larger an eigen–value is in its absolute value, the more pronounced is the curvature of the response surface in the associated direction. Our data revealed that, the model has eigen-values of [λ_1_ = -0.06128758, λ_7_ = -1.00026881 and λ_9_ = -1.02619361]. By applying 1^st^ rule of Myer, our negative eigen- values reflected that the predicted stationary point is maximum. However, based on 2^nd^ rule of Meyer, the two largest eigen-values in their absolute values (1.00026881 and 1.02619361) of our model conferred a pronounced curvature in the directions of two independent variables (X_7_ and X_9_). This finding to a great extent authenticated the results of regression analysis stated that X_7_ and X_9_ exhibited the highest significant effect in linear, quadratic and cross interacted forms on PGase level. Anchored in canonical analysis, the predicted coded stationary point was at {X_1_ = 0.260620063, X_7_ = 0.005901057 and X_9_ = -0.215413876} to achieve a predicted Y of 2.69 μg galacturonic acid.min^-1^. mg^-1^. Besides, the predicted stationary point is clearly positioned inside the explored domain (model constrains).

**Figure 2:**
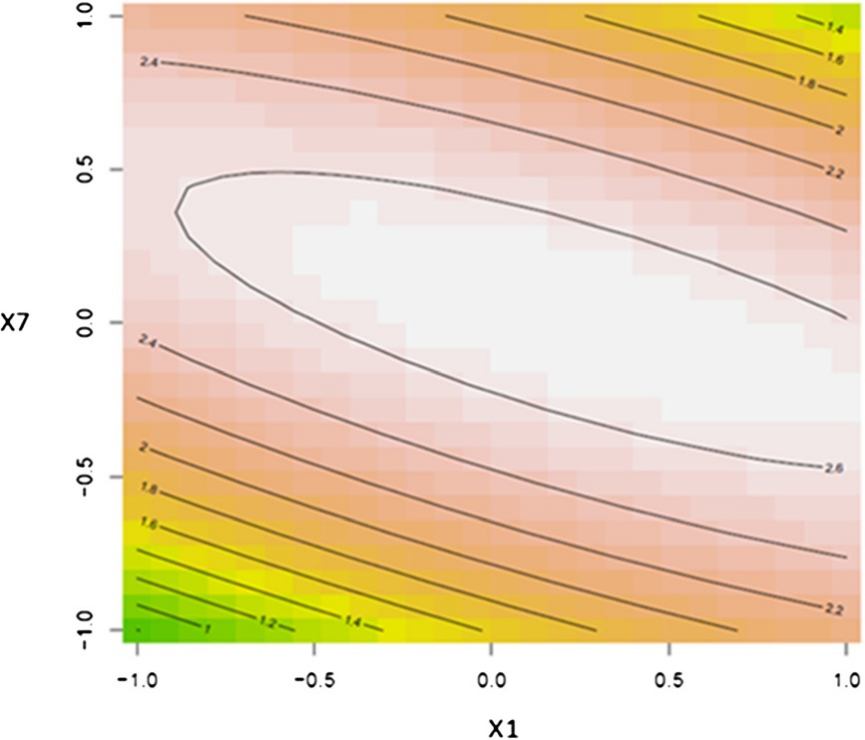
**Contour surface plot for the dependent variable PGase vs. the independent variables; orange peel waste and pH of the production medium.**

**Figure 3:**
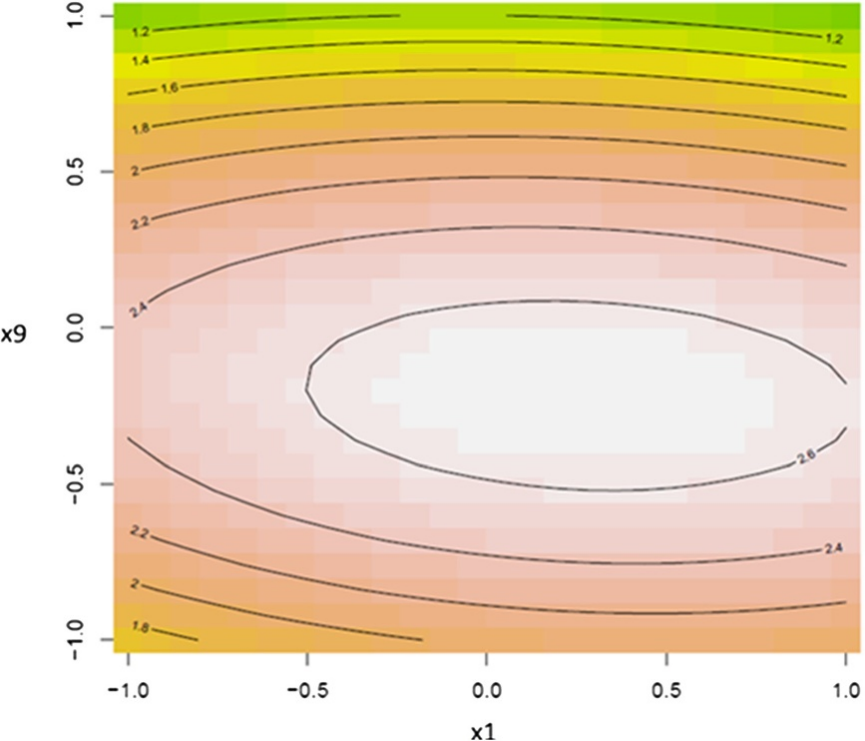
**Contour surface plot for the dependent variable PGase vs. the independent variables; orange peel waste and incubation temperature.**

**Figure 4:**
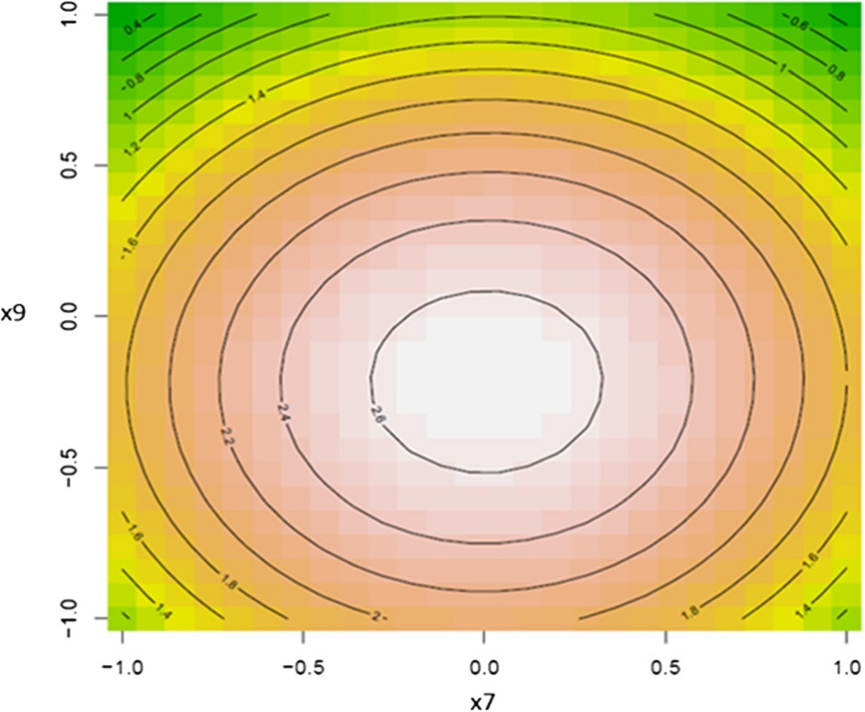
**Contour surface plot for the dependent variable PGase vs. the independent variables; pH of the production medium and incubation temperature.**

To further explore the nature of the response surface at the stationary point, three dimensional contour surface plots were generated. Figures 2, 3 and 4 illustrated the contour surface plots for the response. Typically, the contour surface plots are based on the model, holding one independent variable constant at its optimal level where varying the other two independent variables within the domain. Figure 2 illustrated the response of the dependent variable (PGase) for the optimal level of incubation temperature. The maximal predicted level of PGase 2.69 μg galacturonic acid.min^-1^. mg^-1^ was noticed at levels of 1.76% (w/v) and 8.0 for orange peel waste and pH of the production medium, respectively nearby to the center point of the model. However, the contour surface plot depicted in Figure 3 revealed that the maximal point of PGase at the optimal level for pH of the production medium could be reached at 1.76% (w/v) orange peel waste and 37.8°C. Correspondingly, these predicted levels of both dependent and the independent variables were further evidenced by the contour surface plot illustrated in Figure 4. Concentrations of orange peel waste greater than 1.7% (w/v) did not result in further enhancement in the level of PGase as it was revealed form contour surface plots depicted in Figures 2 and 3. This reflects that a stationary point is achieved at concentrations of orange peel waste exceeding 1.76% (w/v). Conversely higher levels of pH of the production medium and incubation temperature beyond 8.0 and 37.8°C, respectively showed adverse effect on the level of PGase as illustrated from the contour surface plot depicted in Figure 4. These results were verified by the above results of canonical analysis regarding sign and magnitude of model eigen–values.

In addition, validation of the model for PGase production was carried out experimentally by using the aforementioned predicted levels of the independent variables. Experimental data revealed that the adequacy of the model was more or less 100%.

### Optimum pH and temperature for crude PGase

**Figure 5:**
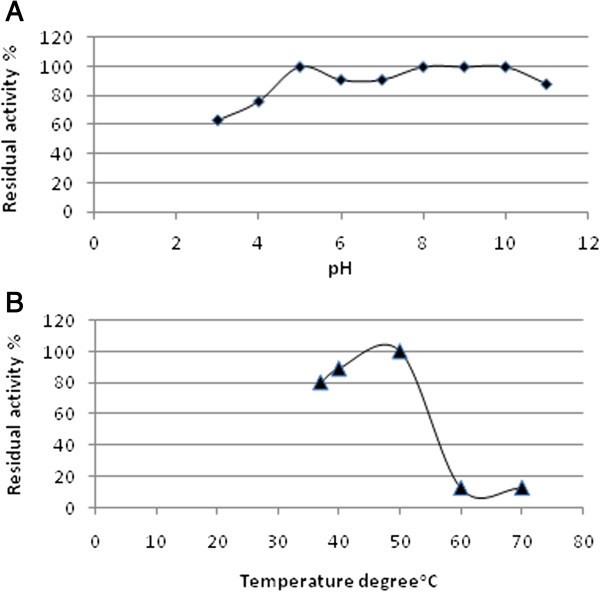
**Effect of different pH(s) [A] and temperature degrees [B] on the activity of PGase SHG10.**

Data revealed that PGase SHG10 showed appreciable level of activity over a wide range of pH (3.0-11.0) (Figure 5a). Whilst, the optimum temperature of enzyme activity was found to be at 50°C (Figure 5b).

### Levels of pectic oligosaccharides

The level of pecic oligosaccharides accumulated in the fermentation broth of this bioprocess was estimated. It was found to be 200 μg galacturonic acid/mL of fermentation broth after 24 hrs. No higher levels of these substances were found in the fermentation broth beyond 24 hrs of incubation.

## Discussion

PGase(s), one member of the family pectinase(s) that have a vast number of industrial applications, still attract the attention of many researchers worldwide. Due to the potential and wide applications of pectinases particularly PGase(s), researchers up till now in worldwide laboratories report the isolation and characterization of novel PGase(s) mainly from fungi and barely from bacteria. Current commercial PGase preparations available in enzymes markets are exclusively derived from fungal species. Normally, PGase (s) of fungal origin have an optima pH ranging from 3.5-5.5. This restricted acidic range of optimal pH greatly confines the extent of fungal PGase (s) utilization. Commercialization of any new enzyme industry is usually constricted to the high cost of production process. Nonetheless, the cost encounter in culture medium accounts for 30-40% of the overall production cost (Bayoumi et al. 2008). From another side, the non-efficiently utilized agro-industrial wastes are accumulated in considerable amounts that could not be abandoned. Furthermore, applying of improper waste disposal methods results in arise of health hazards and environmental problems. Here, the process of PGase production directed by *B. licheniformis* SHG10 was studied thoroughly from the standpoint of low cost effectiveness in conjunction with appreciable yield.

In the course of search for low cost effective medium composition to simultaneously support the growth of *B. licheniformis* SHG10 and induce PGase production, different raw agro-industrial wastes (orange, lemon, pomegranate, banana and artichokes peel wastes) had been introduced separately into the fermentation broth as sole PGase inducers. Our data demonstrated that there exists a profound impact imposed by carbon source on the production of PGase from *B. licheniformis* SHG10. Present finding reveals that raw orange peel waste is a superior PGase inducer comparable with other raw agro-industrial wastes, synthetic citrus pectin and other synthetic carbon sources being tested in this study. Present finding is in disagreement with that of Dey et al. (2011) who reported that synthetic citrus pectin exhibited the best effect as PGase inducer from *Bacillus* sp. AD1 relative to some agro-industrial wastes particularly lemon and orange peel wastes. It was reported that synthetic citrus pectin is the best PGase inducer from *E. aerogenes* NBO2 (Darah et al. 2013). In accordance with our findings, orange peel waste was the best inducer of PGase from *Aspergillus niveus* in submerged state fermentation (Maller et al. 2011). With regard to the impact of wheat bran on PGase productivity from our bacterial strain, data revealed that almost quite similar levels of PGase were obtained upon adding synthetic citrus pectin and wheat bran separately in the fermentation broth. In this respect, the finding of Rehman et al. (2012) is in accordance with our findings. Regarding the impact of synthetic carbon sources alternative to citrus pectin as PGase inducers, none of the tested synthetic carbon sources exhibited stimulatory effect on PGase productivity by *B. licheniformis* SHG10. Findings of Rehman et al. (2012); Jayani et al. (2010) in relation to introduction of glucose, maltose, sucrose, fructose and glycerol into the fermentation broth of *B. licheniformis* KIBGE IB-21 and *B. sphaericus* (MTCC7542), respectively are in good agreement with our finding. Moreover, glucose, xylose, maltose and sucrose as sole PGase inducers were reported to exhibit inhibitory effect on PGase production from *B.firmus* -I-10104 in solid state fermentation (SSF). Glucose and sucrose at a concentration of 1% (w/v) was pointed out to inhibit the PGase productivity from *E. aerogenes* NBO2 (Darah et al. 2013). The noticeable reduction in PGase levels in presence of sugars as a sole carbon source and a PGase inducer could be attributed to the phenomenon of catabolite repression (Ahlawat et al. 2009; Cavalitto et al. 1996; Solís-Pereira et al. 1993).

It was reported that productivity of bacterial and fungal PGase(s) is greatly affected by some mineral salts added to the fermentation broth. To explore the role of these mineral salts on PGase productivity by *B. licheniformis* SHG10, the effect of some mineral salts mentioned above was investigated. Present data revealed that, introduction of MgSO_4_, FeSO_4_, CaCl_2_, KNO_3_ and NaNO_3_ at a concentration of 0.2% (w/v) to the fermentation broth containing synthetic citrus pectin as co-inducer sole carbon source resulted in variable higher appreciable levels of PGase comparable to those levels obtained at zero concentrations of these salts. In accordance with the present finding, KNO_3_ at a concentration of 0.2% (w/v) was the best PGase nitrogen source inducer form *B. licheniformis* growing on the agro-industrial potato peels waste in SSF(Dharmik and Gomashe 2013). In contrast to our finding, neither NaNO_3_ nor KNO_3_ at a concentration of 0.1% (w/v) had shown stimulatory effects on PGase productivity from *B. sphareicus* (MTCC 7542) in citrus pectin containing fermentation broth (Jayani, et al. 2010). Similarly, NaNO_3_ exhibited an inhibitory effect on PGase production from *E. aerogenes* NBO2 (Darah et al. 2013). However, other mineral salts such as NH_4_Cl and (NH_4_)_2_SO_4_ exhibited inhibitory effect on PGase from *B.licheniformis* SHG10. Addition of these two mineral salts separately to the fermentation broth displayed variable levels of PGase from different PGase producers either higher or lower than those obtained at zero concentrations of each salt (Jayani, et al. 2010; Darah et al. 2013; Bayoumi et al. 2008; Dharmik and Gomashe 2013; Kashyap et al. 2003). Concerning yeast extract, also the literature reported varied impact on PGase production from different PGase producers (Rehman et al. 2012; Kapoor et al. 2000).

Low constitutional levels of PGase from *B. licheniformis* SHG10 in presence of peptone, beef or tryptone were detected in fermentation broth lacking any source of pectin either in synthetic form or in raw one. However, other studies reported varied levels of PGase from different PGase producers in fermentation broth containing peptone or tryptone as additional nitrogen sources in presence of pectin source.

In the course of high yield PGase production from *B. licheniformis* SHG10, statistical optimization was applied to maximize the yield of PGase. Roughly identified factors through OVAT, showing a stimulatory effect on PGase production from *B.licheniformis* SHG10 growing on citrus pectin based-basal medium, were studied further through PB. Statistical analysis of data derived from PB revealed that none of the tested mineral salts identified through OVAT; MgSO_4_, KNO_3_, NaNO_3_, CaCl_2_ and FeSO_4_; showed significant impact on PGase productivity in fermentation broth containing orange peel waste instead of synthetic citrus pectin. The agro-industrial orange peel waste seems to be a rich substrate that could co-simultaneously provide the bacterium with all elements needed during the course of bacterial growth and PGase induction as well. This finding greatly alleviates the cost of the PGase production medium.

Substitution of high cost synthetic substrates with raw solid substrates such as agro-industrial wastes in enzyme industry based-bioprocesses is somehow feasible task implying some challenges. The prospect or the scope that a solid waste could cover all needs of a microorganism from organic and inorganic substances mandatory for microbial growth and enzyme induction is considered to be one of the major obstacles in this respect. The greater that essential nutritional elements, mandatory for microbial growth and enzyme induction, exist in suboptimal levels in raw solid substrates, the more indispensable need is to add external supplements in the fermentation broth (Sneath 1986). As a consequence, the cost of the production medium will elevate.

Optimized production of PGase from *B. licheniformis* SHG10 is achievable through using a low concentration of orange peel waste (1.76% w/v) in the fermentation broth as the sole carbon source and the only PGase inducer. This could be attributed to the high content of pectin included in this waste. From another side, the need for low percentage of this waste to support PGase production from *B. licheniformis* SHG10 implies the feasibility of the downstream process in order to remove the remaining undegraded orange peel waste. As a consequence the cost encountered in downstream process would decrease and the overall cost of the process of PGase production would also reduce.

Pertaining to optimal temperature and pH of the production medium required for PGase production, our finding was in a good agreement and disagreement with other findings (Soares et al. 1999; Dey et al. 2011; Maller et al. 2011; de Andrade et al. 2011; Das et al. 2011; Deshmukh et al. 2012). In the context of optima pH for PGase activity, the crude PGase SHG10 was observed to work efficiently under a wide range of pH covering from 3.0 to 11.0 with a slight noticeable decline in the activity at pH 11. Whilst, the optimal activity was confined to the neutral-alkaline scale. In addition, the crude PGase SHG10 showed its activity over a wide range of temperatures ranging from 37°C – 50°C with an optimal activity at 50°C. The requirement of alkaline pH range (8.0 - 11.0) for fulfillment of PGase SHG10 optimal activity features the potential applications of this enzyme in textile processing, degumming of plant bast fibers, treatment of pectic wastewaters, paper making, and coffee and tea fermentations. These two findings concerning optima pH and temperature of our PGase SHG10 imply a clue about the wide range of pH and temperature degrees under which SHG10 PGase could work efficiently. Whereupon, PGase SHG10 would have vast industrial promising applications where each selected combination of pH and temperature degree is a crucial factor to guarantee the success of a certain industrial application. As a consequence, PGase SHG 10 would have a privilege over fungal PGase (s) that have a restricted range of both optima pH and temperature degrees.

Regarding the issue of pectic oligosaccharides production, the literature contains a plethora of chemical methods devoted to synthesize the pectic oligosaccharides that have some reported medical applications (Olano-Martin et al. 2003a,[b]). In this study, data revealed the likelihood of obtaining an appreciated high level of these substances (200 μg galacturonic acid/mL) in the fermentation broth of *B. licheniformis* SHG10 growing on the agro-industrial orange peel waste as a sole carbon source. These recorded levels of pectic oligosaccharides resulted as a consequence of the activity of the PGase produced in the fermentation broth of *B. licheniformis* SHG10 on its complex substrate, orange peel waste. As a matter of fact, the more PGase produced is in the fermentation broth the more liberated pectic oligosaccharides would exist. Therefore, this greatly would necessitate the need for optimizing the process of orange peel waste biodegradation in terms of maximizing pectic oligosaccharides production in the future. However, the nature of the obtained pectic oligosaccharides could be controlled through the possible cascade of pectinase(s) that would be produced by *B. licheniformis* SHG10 growing under the stated conditions. This finding is considered an alternative promising approach implying some challenges towards biosynthesis of pectic oligosaccharides from the standpoints of cost effectiveness, good quality and high yield.

## Conclusions

Present work addresses a cheap rapid biotechnological method to promote PGase and pectic oligosaccharides industries through bioprocessing of the agro-industrial orange peel waste via *B. licheniformis* SHG10. Characteristics of PGase SHG10 concerning the wide range of optima pH greatly confirm its potential biotechnological applications. For the next future work, the authors are planning to further maximize the yield of PGase from *B. licheniformis* SHG10 through a molecular gene cloning approach and improve the physicochemical properties and PGase activity of the enzyme towards pectin-containing substrates through applying directed evolution methodologies. Additionally, the authors are going to optimize the yield of pectic oligosaccharides accumulated in the fermentation broth as a result of the biodegradation of orange peel waste through this bioprocess.

## Methods

### Bacterial strain

*Bacillus licheniformis* strain SHG10 was used in this study as PGase producer. This bacterium was previously isolated from Egyptian soil and identified as *B. licheniformis* SHG10 strain (unpublished data). Its 16S rDNA nucleotide sequence was submitted in the GenBank at NCBI [National Center for Biotechnology Information] under the accession number [GenBank: JN853580]. Moreover, this bacterial strain was deposited in the DSMZ [Leibniz Institute-German Collection of Microorganisms and Cell Cultures] under the accession number [DSM 28096].

### Pectin-containing materials

Different pectin-containing materials were co-utilized in this study as a sole carbon source for the growth of the producer bacterial strain and as an inducer for PGase as well. Pectin-containing materials used in this study included synthetic citrus pectin, wheat bran, orange peel waste, lemon peel waste, banana peel waste, artichokes peel waste and pomegranate peel waste. The last five pectin–containing materials were collected from different sites (e.g.; local Egyptian markets, domestic effluents and agricultural fields). The collected pectin–containing materials were washed with distilled water then were allowed to dry at 60°C for five hrs. After that, the dried pectin–containing materials were prepared in the form of small cut pieces preparation before their incorporation into the fermentation broth. Synthetic citrus pectin was purchased from Sigma –Aldrich Co. While, wheat bran was obtained from flour mills companies in Alexandria, Egypt.

### Media

Peptone yeast broth (Bernhardt et al. 1978) was used to activate the bacterial producer strain. PA medium is peptone yeast broth with 1.5% agar agar. Polygalacturonase core production medium of (Soares et al. 1999) with slight modifications was used in this study. This modified medium was used during the initial steps of the optimization process. The modified PGase core production medium contained the following components in % (w/v): 0.5 g pectin, 0.14 g (NH_4_)_2_SO_4_, 0.6 g K_2_HPO_4_, 0.2 g KH_2_PO_4_, 0.01 g MgSO_4_ and 0.3 g yeast extract unless otherwise stated.

### Inoculum preparation

A fine touch of *B. licheniformis* SHG10 preserved on PA slant was streaked on PA agar and was incubated at 37°C for overnight. One colony was picked to inoculate 20 ml of PY medium in 100 ml Erlenmeyer flask. The inoculated broth was incubated at 37°C with agitation speed of 200 rpm for 4 hrs until the culture OD at 420 nm reached 0.5. Then this growing culture (seed broth) was used to inoculate the fermentation broth (production medium). The inoculum size of the seed broth used to inoculate the fermentation broth was 2%(v/v) unless otherwise stated.

### PGase assay

The PGase activity was assayed by estimating the amount of reducing sugars released under assay conditions. Determination of the amount of released reducing sugars as galacturonic acid was carried out as reported previously (Miller 1959) using 2-hydroxy-3,5-dinitrobenzoic acid [(DNSA), Shanghai Orgpharma Chemical Co., Ltd., China]. Succinctly, the reaction mixture contained 0.5 mL of 0.5% citrus pectin (Sigma-Aldrich Co.) as a substrate (dissolved in 50 mM Tris-HCl, pH7.6) and 0.5 mL crude enzyme (fermentation broth). This mixture was incubated at 37°C for 20 min. After that, the enzymatic reaction was stopped by addition of 1 mL of DNSA followed by boiling for 10 min. Then the final volume was completed to 4 mL by distilled water and the developed color was measured at 540 nm. Control reactions were prepared as mentioned above except that DNSA was added prior addition of the crude enzyme. A standard curve with α-galacturonic acid (Sigma-Aldrich Co.) was established. One unit (arbitrary unit) of enzyme activity was defined as the amount of enzyme that releases one μg of α-galacturonic acid per min from citrus pectin as a substrate in 50 mM Tris-HCl, pH 7.6 at 37°C.

### Protein determination

The protein content of the crude enzyme solution was performed as reported previously using Folin -Lowry reagent (Lowry et al. 1951). A standard curve using bovine serum albumin was established.

### Pectic oligosaccharides determination

Pectic oligosaccharides were determined as reported previously by the method of Miller (1959). Briefly, 0.5 mL of the fermentation broth was added to 1 mL of DNSA. Then, the reaction mixture was boiled for 10 min. After that, the absorbance of the developed color was measured at 540 nm against blank (the same as the reaction except that water was added instead of fermentation broth).

### Optimizing the production of PGase from *B. licheniformis*SHG10

Optimizing the production of PGase from the producer strain was accomplished through a three successive step plan; one variable at a time approach (OVAT), Plackett-Burman design and Box- Behnken design.

### OVAT approach

OVAT was employed in this study in order to screen different independent variables that would either stimulate or inhibit the production of PGase enzyme. This approach is based on changing one variable at a time without studying the interaction among the tested variables. The effect of different agro-industrial wastes (e.g., orange, lemon, banana, artichoke, pomegranate peel wastes and wheat bran) and some synthetic carbon sources (e.g., citrus pectin, tryptone, peptone, beef extract, glucose, maltose, sucrose, xylose, fructose and glycerol) on PGase productivity by *B. licheniformis* was assessed. In addition, the effect of different salts such as NaNO_3_, KNO_3_, NH_4_Cl, CaCl_2_, MgSO_4_ and FeSO_4_ was studied as well. The exact concentrations of the aforementioned tested substances were displayed in Tables 1 and 2.

### Plackett – Burman Design (PBD)

Identifying the significant main key determinants (physicochemical independent parameters) in a bioprocess along with studying the linear effect of these tested variables is achieved by applying a powerful statistical approach namely called Plackett–Burman design that developed by two statisticians Plackett and Burman (1946). In this approach evaluating the linear effect of *N* independent variables on the dependent variable (output of a bioprocess) is tested in a *N + 1* experiment. Normally, each independent variable is studied in two levels -1 and +1; low coded level and high coded level, respectively. The design matrix was generated by a statistical software package Minitab version 15. Here, twenty experimental runs (trials) had been conducted. The following polynomial equation from the first order (Equation 3) was put in order to evaluate the linear effect imposed by the ten tested independent variables on the level of PGase enzyme:
3

Where *Y* is the level of PGase activity, *β*_*0*_ is the model intercept, *X*_*1*_*-X*_*10*_ are the tested independent variables (orange peel waste, NaNO_3_, MgSO_4_, CaCl_2_, FeSO_4_, KNO_3_, pH, inoculum size, incubation temperature and incubation time, respectively) and *β*_*1*_*-β*_*10*_ are the coefficient of the ten tested independent variables. The experimental runs were conducted according to the PBD matrix in 250 ml Erlenmeyer flasks with working volume of 25 ml. All experimental runs were conducted at agitation speed of 150 rpm.

### Box-Behnken design

Three key determinants (orange peel waste percent, pH of the production medium and incubation temperature) identified through PBD had significant effects on the level of PGase. In order to determine the optimal level of each key determinant ((independent variable) along with the maximal level of PGase (dependent variable), a response surface methodology approach was applied here. Box- Behnken design, developed by Box and Behnken (1960), was employed in this study. Fifteen experimental runs (trials) had been conducted. The following polynomial equation from the second order (Equation 4) was set in order to estimate the effect of all possible forms of interactions imposed by the above mentioned three independent variables on the level of PGase enzyme:
4

Where *Y* is the level of PGase activity, *β*_*0*_ is the model intercept, *X*_*1*_*, X*_*7*_ and *X*_*9*_ are the tested independent variables (orange peel waste percent, pH of the production medium, and incubation temperature, respectively), *β*_*1*_*, β*_*7*_ and *β*_*9*_ are linear coefficients, (*β*_*11*_, *β*_*77*_, *β*_*99*_ ) are quadratic coefficients and (*β*_*17,*_*β*_*19*_ , *β*_*79*_) are cross interaction coefficients. For statistical calculations, each independent variable X was coded as Xi according to the Equation 5.
5

where *X*_*i*_ is dimensional coded value of the independent variable, *xi* is the real value of this variable at this coded value, *x*_*o*_ is the real value of this variable at the center point (zero level) and *Δxi* is the step change value. The experimental runs were conducted according to the Box –Behnken matrix in 250 ml Erlenmeyer flasks with a working volume of 25 ml. All experimental runs were conducted at agitation speed of 150 rpm.

### Statistical, canonical analyses and contour plots

RSM package (R Development Core Team 2009), available from the Comprehensive R Archive Network at http://CRAN.R-project.org/package=rsm, was used in this study to carry out multiple regression, canonical analyses and graphing of three dimensional contour surface plots.

### Effect of different pH and temperature on the activity of PGase

Two buffers were used in this study to cover a wide range of pH, 50 mM sodium acetate buffer, pH 3-6 and 50 mM phosphate buffer, pH 7-11. Five different temperatures (37°C, 40°C, 50°C, 60°C and 70°C) were used to test the optimal activity of PGase.
